# A think-aloud study to inform the design of radiograph interpretation practice

**DOI:** 10.1007/s10459-020-09963-0

**Published:** 2020-03-05

**Authors:** Jong-Sung Yoon, Kathy Boutis, Martin R. Pecaric, Nancy R. Fefferman, K. Anders Ericsson, Martin V. Pusic

**Affiliations:** 1 Department of Psychology, University of South Dakota, Vermillion, SD USA; 2 Dept. of Pediatrics, The Hospital for Sick Children, and University of Toronto, Toronto, Canada; 3 Contrail Consulting Services, Toronto, Canada; 4 Department of Radiology, New York University School of Medicine, New York, USA; 5 Department of Psychology, Florida State University, Tallahassee, FL USA; 6 Department of Emergency Medicine, New York University School of Medicine, New York, USA; 7 Division of Learning Analytics, Institute for Innovation in Medical Education, 550 First Avenue, MSB G109, New York, NY 10016 USA

**Keywords:** Education (medical), Radiology, Instructional design, Cognition, Emergency medicine

## Abstract

Models for diagnostic reasoning in radiology have been based on the observed behaviors of experienced radiologists but have not directly focused on the thought processes of novices as they improve their accuracy of image interpretation. By collecting think-aloud verbal reports, the current study was designed to investigate differences in specific thought processes between medical students (novices) as they learn and radiologists (experts), so that we can better design future instructional environments. Seven medical students and four physicians with radiology training were asked to interpret and diagnose pediatric elbow radiographs where fracture is suspected. After reporting their diagnosis of a case, they were given immediate feedback. Participants were asked to verbalize their thoughts while completing the diagnosis and while they reflected on the provided feedback. The protocol analysis of their verbalizations showed that participants used some combination of four processes to interpret the case: *gestalt interpretation, purposeful search, rule application,* and *reasoning from a prior case.* All types of processes except reasoning from a prior case were applied significantly more frequently by experts. Further, gestalt interpretation was used with higher frequency in abnormal cases while purposeful search was used more often for normal cases. Our assessment of processes could help guide the design of instructional environments with well-curated image banks and analytics to facilitate the novice’s journey to expertise in image interpretation.

## Introduction

Diagnostic images play an important role in everyday medical practice. Radiographs, in particular, are one of the most commonly ordered image-based tests (Boutis et al. 2019). Since interpretation errors are a potential threat to patient safety (Graber et al. 2012) considerable resources are invested in training clinicians to high levels of performance. However, development of expertise by bedside exposure to radiograph cases is often insufficient (Dixon 2015; Reeder et al. 2004; Ryan et al. 2004; Trainor and Krug 2000) and does not offer opportunities for individualized training to master particular weaknesses in diagnostic accuracy. Importantly, there is an incomplete understanding of how the cognitive processes mediating superior performance in radiograph interpretation develop (Gegenfurtner et al. 2017).

Ericsson and colleagues have shown that a key enabler for attaining the highest levels of expertise is engaging in deliberate practice. Deliberate practice supports the development of refined mental representations which allow the expert clinician to engage in elaborate reasoning strategies and information processing (Ericsson and Pool 2016). Since radiographs from patients with verified diagnoses can be collected and presented using digital libraries, deliberate practice of radiograph interpretation is possible. Specifically, one can include in the digital library hundreds of images to ensure representation of necessary exemplars of all relevant types, creating a spectrum of exposure that would otherwise take years to experience directly in the clinical setting (Pusic et al. 2011; Ericsson 2015). Further, the digital environment can authentically capture the situation of making a diagnosis of patient’s radiographs by asking the learner to judge the existence and location of abnormalities on an unmarked image (Pecaric et al. 2017). Immediately following the diagnosis, the learner can receive feedback on the appropriateness of their response and thus can learn from errors with every case encounter (Ericsson 2004, 2015).

Digital learning platforms can also be useful in capturing the mental representations of novices and experts; comparing these representations can allow us to consider the best instructional path from novice to expert (Boutis et al. 2010; Pecaric et al. 2017). For example, specific participant behavioural data like time spent reviewing a case, number of views examined, and presence of localization errors have demonstrated differences between novice and expert patterns of radiograph interpretation (Pecaric et al. 2017). This data can be used in learning algorithms so as to promote meta-cognition, self-directed learning, and education management (Pecaric et al. 2017; Plass et al. 2013).

The most effective skill acquisition requires objective *intermediate* goals for improving performance determined by the learners’ current mental representation as well as the next possible improvements, describing a path toward expert performance. Detailed learning activities are then designed to allow the learners to improve their representations and gradually attain the assigned goal. This contrasts with other types of practice where many hours can be spent in the activity but without a guiding model of learning to suggest which sub-activities are appropriate to the specific skill being developed (Ericsson 2018a). Therefore, to optimize the journey of learning radiograph interpretation, it would be beneficial to better understand the intermediary process of learning radiograph interpretation by capturing cognitive processes as this skill develops.

Cognitive models of radiograph interpretation by experts have been well elucidated. Kundel et al. proposed a four-part cognitive framework (Kundel et al. 1978; Kundel 2000). The radiologist first rapidly orients to the image, establishing its overall properties (type, quality, perspective). Next s/he scans the image to detect features. Candidate features are each considered and a decision is made as to the feature’s significance—pathology or not. Once a feature’s relevance is decided, the scan is resumed to find the next feature. This process is continued iteratively by the clinician until they are satisfied that all relevant features have been identified and considered. The Kundel framework is based on eye-tracking studies of participants of varying levels of expertise, determining their response latencies and eye-fixations during interpretation of radiographs with known properties. However, few if any of these studies were carried out in a learning context, and thus how an immature cognitive model of visual diagnosis progresses to a refined one remains relatively unknown (Gegenfurtner et al. 2017; Kundel 2000). Think aloud methods have been used to describe thoughts related to expertise in medical image interpretation of diagnostic medical images (Azevedo et al. 2007; Crowley et al. 2003; Lesgold et al. 1988; Sibbald and de Bruin 2012; van der Gijp et al. 2015, Morita et al. 2008). Thus, this approach could be used as a referent for the learning of radiograph interpretation, allowing better understanding of the development of radiograph interpretation expertise.

This paper is focused on how expert diagnostic performance is acquired through learning. In particular, we took advantage of the protocol analysis of think-aloud reports and an established digital platform that presented diagnostic images with feedback to determine how mental representations of visual diagnosis in medical students are gradually refined to match the representations of experts. Such a model could be the basis of more effective deliberate practice of this important skill.

## Methods

We present an expert-novice comparison of the act of radiograph interpretation, where the novices’ thought processes are expected to evolve as they actively learn while the experts’ thought processes provide a point of reference. The thought processes were collected using think aloud verbal protocols. Further insight was obtained from numerical process data from the digital learning environment.

### Think aloud verbal protocols

Think-aloud is a research method in which participants give verbal expression to their thoughts as they focus on completing a task. It has been shown that participants are able to think aloud without influencing the accuracy of performance as compared to a silent traditional condition of performing the same tasks (Fox et al. 2011). Thinking-aloud is considered distinct from introspection since thinking aloud involves only focusing on a challenging task while concurrently giving verbal expression to thoughts entering attention (Ericsson and Fox 2011). It has been applied in medical education contexts as a means of uncovering clinical reasoning (Pinnock et al. 2015; Smeets et al. 2019). Think-aloud protocols may also be well-suited to better understanding expertise in radiograph interpretation since this is a skill of intermediate cognitive difficulty and involves sequential cognitive (thought) processes (Ericsson 2018a). Further, think-aloud protocols allow investigators to codify responses and explicate cognitive processes that would generate the verbalized information (Ericsson 2018a).

Ericsson and Simon’s (1993) model assumes that verbal reports may be incomplete because participants are advised explicitly that verbalization of thoughts should be secondary to performance of the main task. As such, a complementary method for collecting information of participants’ thinking (Ericsson and Simon 1993) involves asking the participants after the completion of a task to recall as much of their thinking as possible starting with the first thought that they can remember (retrospective verbal reports). This procedure is quite different from another procedure of having participants “think aloud” after the end of the completed experiment while the participants are shown videos of their behavior generated during the experiment (Sibbald and de Bruin 2012). The current study adopted the recommended instructions and procedures for protocol analysis, which includes initial instruction, warm-up procedures, reminders only to keep talking, and directing the participant to focus on the presented task rather than introspect and describe their thought processes. Think aloud has been shown to not change the course of the thought processes, minimizing any observable effect on accuracy of performance (Ericsson and Simon 1993; Fox et al. 2011).

#### Overall study procedure

We presented a series of pediatric elbow radiographs to seven medical students (novices) and three senior radiology residents and one attending-level pediatric radiologist (experts) We collected think-aloud verbal reports of participants for each image during their diagnostic process. The system provided immediate corrective feedback on their interpretation which allowed novices to learn with each case. While experts might also learn from the feedback, their learning would be much less. The intention was to provide an expert-level (non-learning) comparison for the presumed learning processes in the novices.

After explaining the process of think-aloud research, an investigator-moderator was present but had only a passive role and did not prompt or encourage explanation. Verbal reports were coded for themes that reflected different types of thought processes of participants, based partly on Kundel’s previously published cognitive framework for visual diagnosis: holistic/gestalt impression, searching, and prior knowledge/pattern recognition (Kundel 2000; Kundel and John Wright 1969; Kundel 2007) but also anticipating there would be learning processes. Additionally, data collected digitally were analyzed for diagnostic accuracy, respondent confidence and time spent on each case. Comparisons were then made between novices and the more experienced practitioners.

### Diagnostic images

**Fig. 1:**
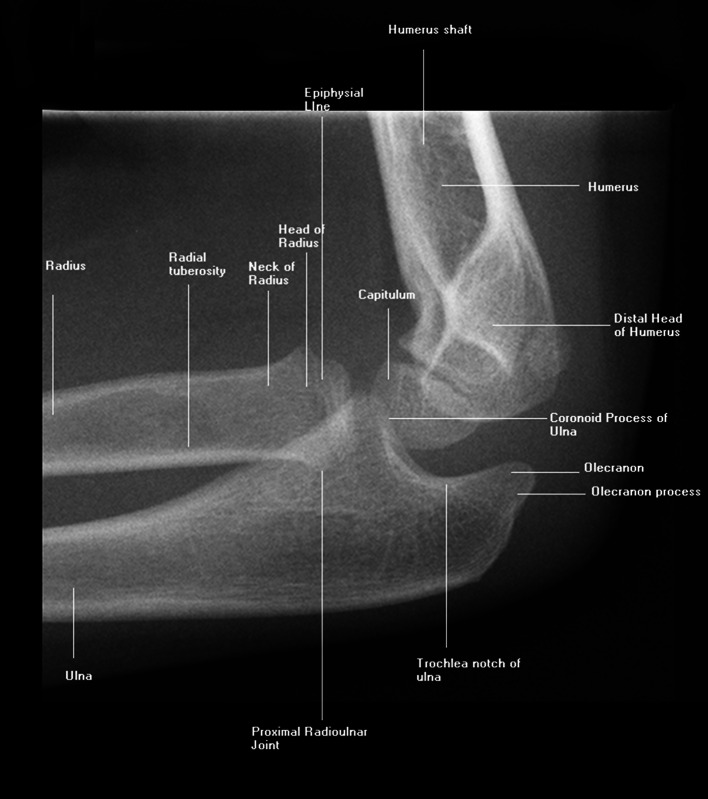
Typical pediatric elbow radiograph. The radiologic anatomy of a pediatric elbow showing some of the features that would be considered in searching for an abnormality such as a fracture

We chose pediatric elbow radiographs taken in the setting of possible fracture as the subject of this study because this is a moderately difficult cognitive task among medical students (Fig. 1), which is ideal for think-aloud research methods (Ericsson and Simon 1993). It is also representative of an authentic clinical task and amenable to practice with immediate feedback in an on-line environment (Boutis et al. 2019), and thus interpretation of these images is likely to induce variation between participants and be free of ceiling effects. Specific concepts relevant to the diagnostic interpretation of these images are listed in Table 1.

**Table 1:** Pediatric elbow radiograph concepts

Concept	Definition	Implication
Normal growth plate	Growth plates are cartilaginous structures that appear as radiolucent lines on radiographs	May mimic fractures
Fat pads	Fat pads appear both anteriorly and posteriorly at the distal humerus. The posterior fat pad is normally not visible; if seen, a fracture is generally presumed present. Anterior fat pads can be seen under normal circumstances	Specific knowledge or experience is required to first identify the fat pads and second to know that one signifies pathology and the other may not
Anterior humeral line	Fractures of the distal humerus can be subtly displaced; one analytical method of detecting such fractures is to draw an imaginary line down the anterior aspect of the humerus and then determine the degree to which it intersects the distal humerus	This technique is context specific. It would be expected to be available to experts but not novices.
Mid-radial line	Dislocation of the radius may be detected by comparing the alignment of the radius with the bone with which it articulates with malalignment indicating abnormality	This is another analytical technique expected to distinguish experts from novices

### Selection of images and digital preparation

In a previous study, we collected 285 pediatric elbow radiographs taken for the purpose of excluding fracture and this set included a range of diagnoses, including normal examples (Boutis et al. 2019; see Table 4 in “Appendix”). Cases consist of the standard set of two images (AP and lateral) and included a brief clinical history based on the imaging requisition. Each case was marked-up a priori by a radiologist using graphics that highlight the area of abnormality and then embedded into a template generated using the Flash integrated development environment (Adobe Systems Inc., San Jose, CA). The presentation software is described in a recent publication (Boutis et al. 2019) and was shown to be effective in improving learner interpretation accuracy, even within 20 cases (Boutis et al. 2019: Fig. 3).

These radiographs were calibrated for difficulty-level using classical test theory analyses (AERA, 1985). We classified cases into three sets: (1) easy (mean *p* = .85; i.e., 85% accuracy from pilot testing with a mixed group), (2) intermediate (mean *p* = .70), and 3) difficult (mean *p* = .41). The current study selected cases for each difficulty level. In particular, there were 29 easy cases available only to medical students, 20 intermediate cases available to both the medical students and the experts, and 28 difficult cases available only to the experts. Thus, the intermediate cases were intended to be “difficult” items for the medical students but these same cases were expected to be “easy” items for the experts. This was done to avoid floor or ceiling effects due to items being too easy or hard for either group.

The set of cases consisted of the same number of normal to abnormal and the same equal ratio of difficult to easy cases, keeping in mind that the difficulty levels differed for each skill group as outlined above. The cases were presented in a fixed order to all participants and the participants completed as many cases as possible during a 60-min session. Examples are shown in the “Appendix”. The software tracked their progress through the cases and recorded their responses. The participants were not provided with any information about the proportion of normal to abnormal cases or types of pathology in advance of participation.

### Study participants

Participants were recruited in the Department of Radiology at New York University from March to May 2015. The “novices” were third-year medical students who were on a radiology rotation. The experts in our study were third and fourth year radiology residents who had completed two pediatric radiology rotations, and one attending-level radiologist. The study activity was within the general educational objectives of the medical student rotation but they had not had explicit instruction in the reading of pediatric elbow radiographs. Participants were recruited through a general email solicitation and were paid $50 in appreciation of completing the study procedures. The study was approved by the NYU School of Medicine Institutional Review Board.

### Study procedures

One participant at a time completed the study protocol. After completing informed consent, participants were seated before a computer. They were instructed on how to provide both concurrent and retrospective think aloud (verbal) reports (Ericsson and Simon 1993; Fox et al. 2011). During the instruction (Ericsson and Simon 1993; Fox et al. 2011), the participants practiced with several “think-aloud” warm-up tasks (e.g., a simple arithmetic question) where it is relatively easy to think aloud. The think aloud instruction was given by a research associate trained in these methods.The operation of the computer software was demonstrated by having the participant complete two “warm-up” radiograph cases.

**Fig. 2:**
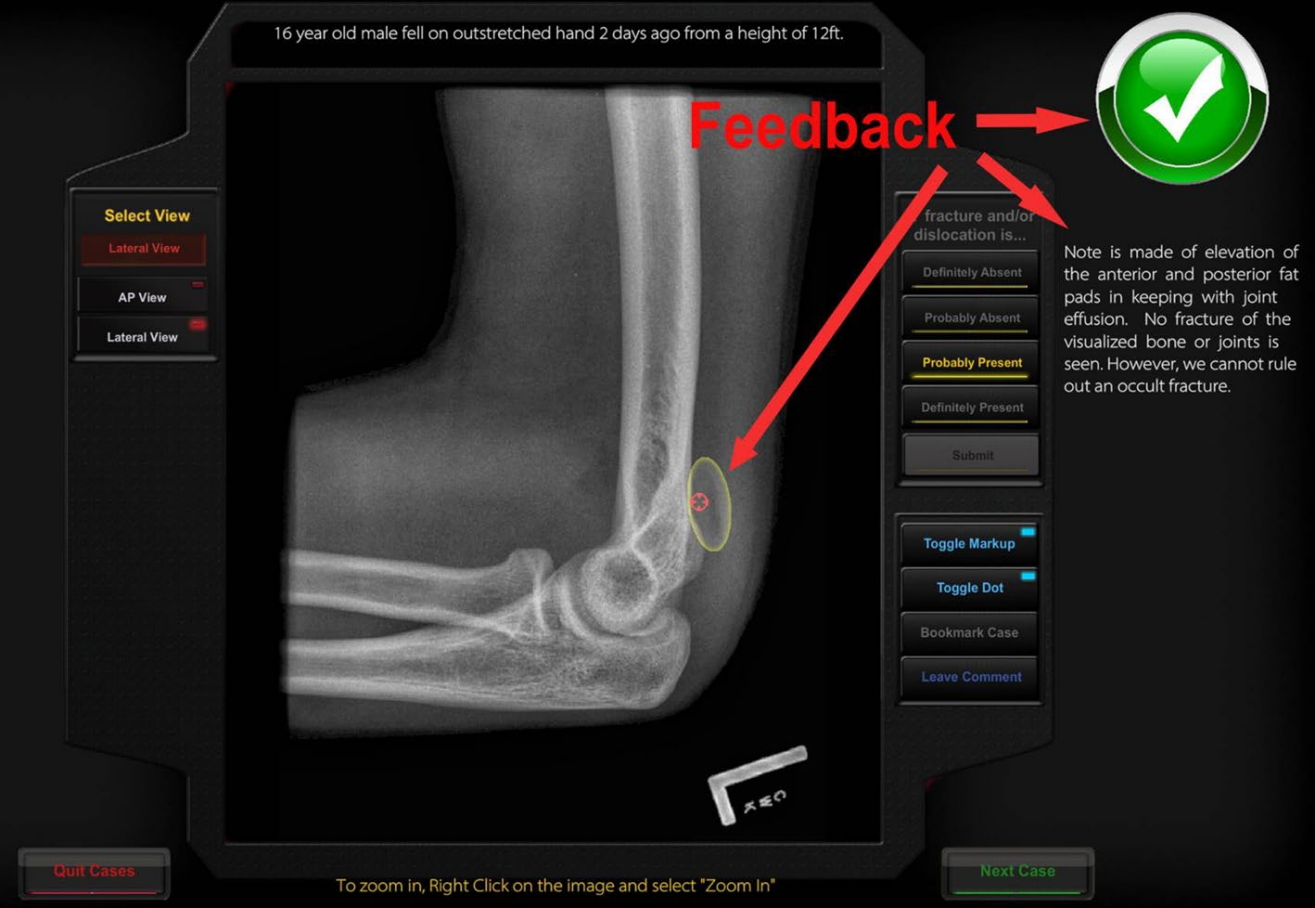
A screen capture from the ImageSim learning system used in the study. Shown is the feedback page demonstrating: **a** the learner’s assignation of where the fracture lies (red marker) which would have been placed by the learner on a prior screen, on an otherwise unmarked radiograph; **b** three forms of feedback including overall correctness (green checkmark), text of radiologist report, and yellow target area pre-assigned by an expert radiologist. (Color figure online)

Participants did not receive any study-specific training on how to interpret pediatric elbow radiographs prior to starting the study cases. They then diagnosed a series of pediatric elbow cases presented using the digital platform described above. Case interaction included a screen listing the presenting complaint and an unmarked radiograph of the patient. Clicking the appropriate button took the participant to one of the standard radiograph views. The participant was able to access any view as (s)he wished. No time limitation was imposed during participation. When ready, the participant declared the case either clinically “normal” or “abnormal” with modifiers (“Probably”/”Definitely”) suggesting how confident they were in the diagnosis. If the answer was that the radiograph is “abnormal,” the participant then marked the radiograph, using a cursor to indicate where they thought the abnormality was located. They then committed to their answer by clicking a “Submit” button. While performing each case, participants were asked to concurrently verbalize their thoughts out loud (i.e., think aloud). If there was a period of silence (around 10 s), the experimenter reminded participants to think aloud by saying “Keep talking” as recommend in Ericsson and Simon (1993). After the diagnosis of each case, but before clicking the “Submit” button, the participants were asked to retrospectively verbalize their thoughts again as much as possible (i.e., retrospective verbal report). After participants clicked the “Submit” button the system provided instantaneous feedback, including a visual overlay indicating the region of abnormality (if any) and presentation of the entire official radiology report. An example of the feedback screen is shown in Fig. 2. Participants also verbalized their thoughts while considering the case feedback. Once the participant had considered this information, they went on to the next case.

Each participant session lasted around 60 min during which the participants were to diagnose a minimum of 12 radiograph cases. To ensure even distribution of case types, we presented cases in blocks of 4 cases, where two normal cases (one easy and one difficult) and two abnormal cases (one easy and one difficult) were presented in random order. The abnormal diagnoses were chosen to represent findings that could be detected through either pattern recognition (e.g. posterior elbow effusion) or analytical means (e.g. radius dislocation confirmation through rule application).

All of the participants’ utterances were audio- recorded for subsequent transcription. Further, we recorded each screen change in a time-stamped manner along with the coordinates of their localizations of suspected fractures on the images.

### Data analyses

**Fig. 3:**
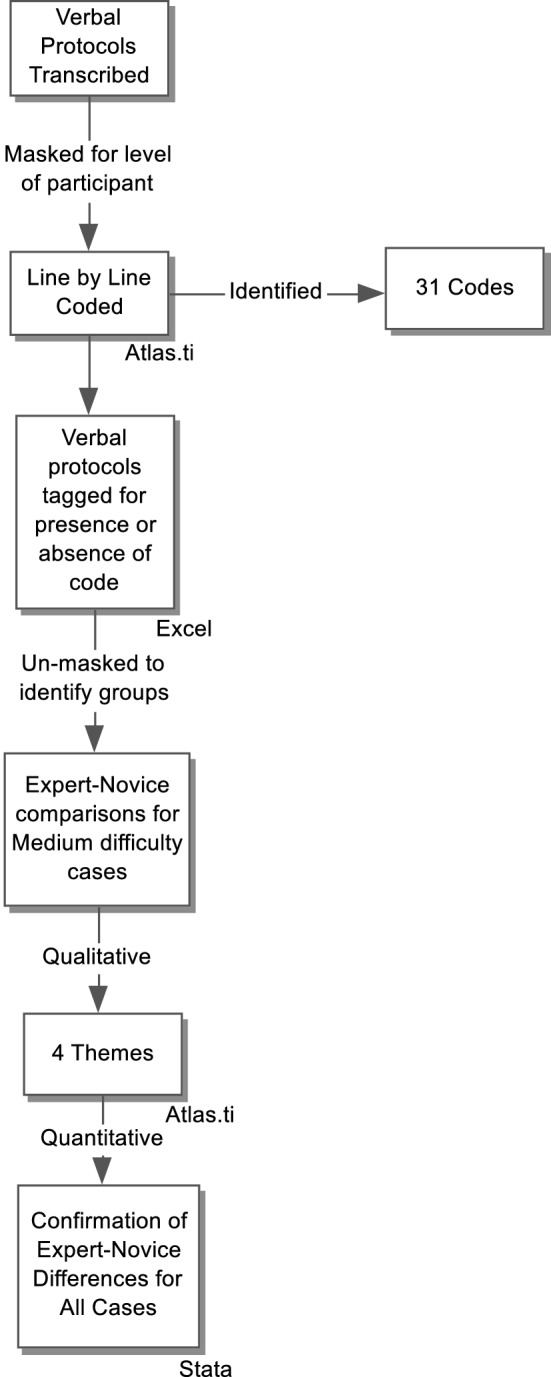
Mixed method analysis of verbal protocol and log file data

Verbal protocol analysis was conducted by a research associate trained to collect and analyze verbal reports. We analyzed all verbal reports as a corpus using Atlas.ti (Scientific Software Development GmbH, Berlin, Germany; Version 6.0) informed by the framework that includes holistic/gestalt impression, searching, and prior knowledge/pattern recognition, as previously described in research that examined the approach to visual diagnosis of radiographic images (e.g. Kundel et al. 1978; Mello-Thomas et al. 2005; Wood et al. 2013). Importantly, we also left ourselves open to identifying new themes (Fig. 3) including expressions of learning from the cases. The reports were line-by-line coded and analyzed by a clinician who was both expert in the subject matter and in thematic analysis (MP). The protocols were analyzed blind to the expertise level of the participant and to the correctness of the interpretation. All images were available to the coder to enable full inferences. The coding was done in two waves: MP developed an initial list of codes analyzing transcripts until no new codes were emerging (approximately 100 cases). This list of codes was discussed with the investigative team with new ideas emerging. MP then returned to the transcripts and started again and re-coded every case with the new extended list of codes. The codes were synthesized into overall themes. MP then went back to each case verbal report and tagged it as to whether one or more of the themes applied to that instance. After unblinding as to which verbal report belonged to which level of participant, we performed between-group numerical comparisons. In comparing novices to experts, the direction and statistical significance of the findings was the same whether analyzed by nonparametric Chi square, cluster-adjusted logistic regression or t-tests. We chose, for ease of interpretability, to report univariate comparisons as t-tests with 95% confidence intervals of differences. Multivariate tests are reported as adjusted Odds Ratios with their 95% confidence intervals from the cluster-adjusted logistic regression.

A priori, we planned to record 100 cases by novices and experts. Sample size was titrated to a qualitative end-point, namely saturation of codes on verbal reports across case types. Each case completed by a participant was considered one item. Normal items were scored dichotomously depending on the match between the participant’s response and the original radiology report. Abnormal items were scored correct if the participant had both classified it as abnormal and indicated the correct region of abnormality on at least one of the images of the case. Participant “certainty” was measured as the percent of responses reported as “definitely normal or definitely abnormal” versus “probably normal or probably abnormal” (Pusic et al. 2015). Time on case was measured from the time a case was initiated to the time the next case was initiated.

## Results

### Study participants

We enrolled seven medical students (novices), three senior residents and one attending-level radiologist (our experts). A fifth attending-level radiologist also completed a session but their data was lost due to a tape recording error. Verbal protocols were collected for 191 cases, 102 by novices and 89 by the experts. Novices completed a median of 15 cases (IQR 13, 17.5) and experts 20.5 (IQR 20, 22.75).

### Validity checks

As would be anticipated, there were clear differences in performance between the relative expert and novice groups. Even though the experts did more difficult cases overall, they had better accuracy (65/89; 73% cases answered correctly vs. 37/102; 36% (difference 37%; 95% CI 23%, 50%). Experts also completed more cases in the allotted time (median 20.5 cases vs. 15), taking an average of 93 s per case, compared with 126 s for the novices. The expert group reported being more confident in their diagnoses, choosing the “Definitely” qualifier, in preference to “Probably” 50% of the time, significantly more frequently than did the novices: 17% (Difference 33%; 95% CI 23%, 48%).

### Verbal protocol analysis

Coding of the verbal protocol transcripts yielded 31 codes (see Table 5 in “Appendix”). The number of verbal reports was sufficient to achieve saturation with no new codes emerging after the first 50 cases were coded for each group. Approximately half of the codes aligned with recognized best practices in reading pediatric elbow radiographs (Iyer et al. 2012; Jacoby 2007). Several codes dealt with the learning context including expressions of uncertainty, references to the feedback received and the decision-making process.

### Main outcome: themes

We identified four themes within the verbal protocols: gestalt, purposeful search, rule application and reasoning from a prior case. A typical example of each theme is presented in Table 2 and descriptive numerical analyses are summarized in Table 3. Below we describe each theme using analyses from both the verbal protocol analyses and the complementary quantitative analyses. Quotations in support of each qualitative theme are supplied in the “Appendix” along with representative radiologic images (Tables 6, 7, 8, 9).

**Table 2:** Themes resulting from verbal protocol analysis

Theme	Description from study data	Typical example	Educational implication
Gestalt	Immediately within first consideration of the radiograph the individual recognizes a fracture or key abnormality. More common in experts	Supracondylar fracture with a discontinuity of the cortex	Bottom-up processing; learning requires exposure, with feedback, to a large number of examples and non-examples
Purposeful search	Successive radiograph features are considered in turn until the search space is exhausted. Novices use vague language and are less likely to use semantic qualifiers or identify pertinent negatives	Normal radiograph where there are growth plates to be distinguished from fractures	Visual vocabulary needs development both for labelling and naming features of the normal environment and for identifying the range of variation in normal appearance. Identification of semantic qualifiers and pertinent negatives is an additional learning task
Rule application	Explicit procedures are invoked to clarify decision-making about a borderline or occult disease process. More common in experts. Experts more accurate in cases where they invoke a rule	The Mid-Radial line would normally intersect the capitellum; when it does not then there is likely a proximal dislocation of the radius	Relevant rules and recipes need to be learned as well as when they are properly applied
Reasoning from a prior case	Novices were likely to carry forward new knowledge from the feedback given on prior cases. Novices had a trend towards being more successful when using this strategy though the comparison is underpowered	A key feature on some radiographs is the appearance of the posterior fat pad. In considering such a case, the individual reasons from the remembered appearance of the fat pad on a prior case for which they know the gold standard classification	The sequence of presentation of cases is important for the development of novice’s internal representations of normal and abnormal. Perhaps more prototypical cases should be presented early

**Table 3:** Proportion of cases, SDs, 95% Cis, and t-values by verbal protocol analysis theme

Verbal reports	Case	Expertise		Mean difference	95% CI of	t-value
	difficulty	Student	Expert		difference	
Immediate Gestalt	All^a^ Intermediate^b^	.18 (.38) .21 (.41)	.43 (.50) .45 (.50)	− .25 − .24	[− .38, − .12] [− .43, − .06]	− 3.92** − 2.60*
Purposeful search	All Intermediate	.09 (.29) .10 (.30)	.65 (.48) .68 (.47)	− .56 − .58	[− .67, − .45] [− .74, − .43]	− 10.02** − 7.39**
Rule application	All Intermediate	.14 (.35) .10 (.30)	.47 (.50) .59 (.50)	− .33 − .49	[− .46, − .21] [− .66, − .33]	− 5.42** − 6.02**
Reasoning from a prior case	All Intermediate	.20 (.41) .19 (.40)	.07 (.25) .07 (.25)	.13 .12	[.04, .24] [− .01, .26]	2.78** 1.78

		Case type				
		Normal	Abnormal			
Immediate Gestalt	All^c^ Intermediate^d^	.18 (.39) .11 (.32)	.40 (.49) .51 (.50)	− .22 − .39	[− .35, − .09] [− .57, − .22]	− 3.40** − 4.40**
Purposeful search	All Intermediate	.42 (.50) .47 (.50)	.28 (.45) .25 (.44)	.14 .21	[− .00, .27] [.02, .40]	1.97† 2.16*
Rule application	All Intermediate	.33 (.48) .40 (.49)	.26 (.44) .27 (.45)	.08 .12	[− .05, .21] [− .07, .31]	1.16 1.24
Reasoning from a prior case	All Intermediate	.17 (.38) .19 (.39)	.12 (.33) .10 (.30)	.05 .09	[− .05, .15] [− .05, .23]	.95 1.23

^a^*N* = 102, 89 (Student, Expert); ^b^*N* = 52, 44 (Student, Expert); ^c^*N* =  75, 116 (Normal, Abnormal), ^d^*N* = 43, 51 (Normal, Abnormal). † *p* = .05. ** *p* < .01, *p < .05

**Fig. 4:**
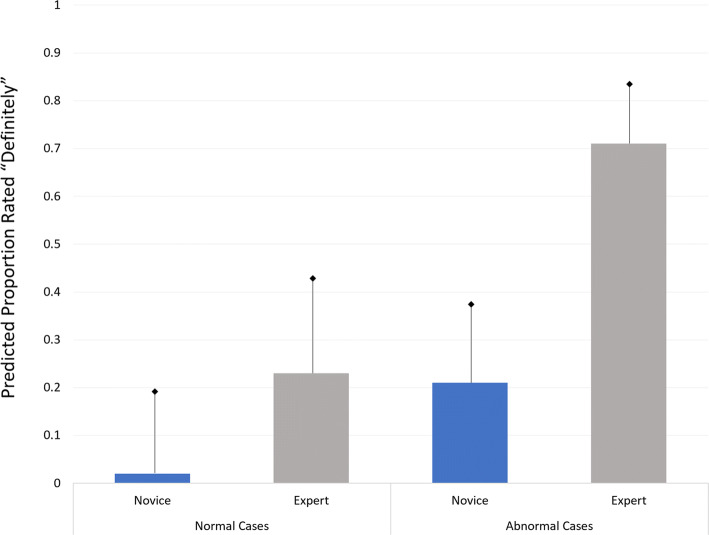
Certainty by expertise level. As expected, experts were found to be more certain in their diagnoses, especially for abnormal cases. Plot shows predicted margins for a logistic regression model predicting use of the qualifier “Definitely” based on case type (Normal, Abnormal) and level of expertise. Whisker shows upper bound of 95% CI

1. *Immediate Gestalt Diagnosis* In a number of the abnormal cases the participants recognized the fracture or the key feature within the first verbalized consideration of the case. They typically did this without describing a process for arriving at the diagnosis. Immediately after reading the history, the diagnosis was the first visual feature described. The Gestalt diagnosis appeared more frequently in experts (M = .43, SD = .50) than in novices (M = .18, SD = .38); difference = .25, 95% CI [.12, .38], *t*(189) = 3.92. The Gestalt diagnosis was also more frequently reported in abnormal cases (M = .40, SD = .49) than in normal cases (M = .18, SD = .39), difference = .22, 95% CI [.09, .35], *t*(92) = 3.40. Adjusted for expertise level, cases where gestalt was seen were no more likely to have been judged accurately (OR: .84; 95% CI .22, 3.15).
2. *Purposeful Search* Overall, in purposeful search, the participant considered features in turn and typically described reasons as to why or why not the feature should be considered a fracture. The participant would list off the features they were considering with varying degrees of specificity both in terms of granularity (e.g. “the alignment is off” versus “the radius is dorsally displaced”) and specialized vocabulary (e.g. “ossification center”, “apophysis”). The responses also varied in the use of “semantic qualifiers” such as anterior/posterior, medial/lateral, small/large effusion, degree of displacement or maturity. Pertinent negatives were sometimes included in the description of the search. There was considerable variability in the actual sequence of features considered both between individuals and between cases done by the same individual. Neither experts nor novices had a stereotypic order with which they considered features of the radiograph. Experts were more likely to use semantic qualifiers. They were also more likely to use specialized anatomical language and to list pertinent negatives. For the quantitative analysis, we tagged a search as being purposeful if it contained one of: specialized vocabulary; pertinent negatives; or semantic qualifiers. Purposeful search was more frequently reported in experts (M = .65, SD = .48) than in novices (M = .09, SD = .29); difference = .56, 95% CI [.45, .67], *t*(189) = 10.02. It was more frequently observed in normal cases (M = .42, SD = .50) than in abnormal (M = .28, SD = .45); difference = .14, 95% CI [.00, .27], *t*(92) = 1.97. Adjusted for expertise level, cases where purposeful search was invoked were no more likely to have been judged accurately (OR: 1.82; 95% CI .21, 15.6).
  An important component in the purposeful search concerns the manner of terminating the search. That is, when participants had found a feature that they determined to be abnormal, they often terminated the search confidently. In normal cases, the end of the search was less well defined and there were frequent expressions of uncertainty. This is consistent with the ratings of certainty. Cases declared “Normal” by the participant were overall more likely to be qualified with “Probably” (80%) than were those declared “Abnormal” (59%; diff 21%; 95% CI Diff: 7.7%, 34%) and this effect was more pronounced for experts than for novices (see Fig. 4 in “Appendix”)
3. *Rule Application* As would be expected, rule application was more frequently reported by experts (M = .47, SD = .50) than by novices (M = .14, SD = .35); difference = .33, 95% CI [.21,.46], t(189) = 5.42. It remained significantly greater in experts when only the medium cases are considered (difference = .49, 95% CI [.33,.66]). There was no difference in the frequency of rule application between normal and abnormal cases. Experts appeared to invoke rules selectively, in cases with a high probability of the rule being directly applicable. This was supported by a logistic regression analysis using all cases. With a dependent variable of case accuracy, expert level (novice/expert) and rule application (yes/no) interacted such that the interaction term was statistically significant (OR 4.5; 95% CI 1.6, 12.8) suggesting the experts functioned better when they applied the rule (predicted marginal accuracy 83% with a rule vs. 63% without). The novices had the inverse relationship, being less accurate on the rare occasions when they applied a rule (29% with rule vs. 38%).
4. *Reasoning from a prior case* During consideration of a given case, novices (M = .20, SD = .41) were more likely than experts (M = .07, SD = .25) to mention prior cases (difference = .13, 95% CI [.04, .24], t(189) = 2.78). Most of the utterances that dealt with prior cases had to do with the novice carrying forward new knowledge from the feedback given on prior cases encountered during the testing session (Table 9 in “Appendix”). Analysis restricted to only the intermediate cases showed the same direction of result, but did not reach statistical significance (difference = .12, 95% CI [-.01, .26]). There was no significant difference in the frequency of reasoning from a prior case between normal and abnormal cases. Amongst all 101 novice cases, when a prior case was mentioned accuracy was 10/21 (47.6%) compared with 27/80 (33.8%) when it was not (difference = 13.9%; 95%CI − 9.6%, 37.3%). There were too few prior case mentions (N = 6) amongst experts to perform a comparable analysis.

## Discussion

In this study of novices and experts who participated in a think-aloud study during learning, we identified four main cognitive processes mediating the interpretation of pediatric elbow radiographs. These processes were *gestalt interpretation*, *purposeful search*, *rule application*, and *reasoning from a prior case*. They were differentially applied by expertise level, with all except reasoning from a prior case being applied more frequently by our experts. Gestalt interpretation was used with more frequency in abnormal cases while purposeful search was used more often for normal cases. Overall, these findings both extend current theoretical frameworks of expertise and provide insights into the process of visual skill development that can inform radiograph interpretation teaching interventions.

Identifying the differences in the reliance on these processes between novices and experts can shed light on why expert performance in diagnostic interpretation is so difficult to attain (Ericsson and Smith 1991). The protocol analysis of think-aloud reports revealed at least four different learned cognitive processes that mediated diagnostic performance, with each being triggered by characteristics of the specific case. Each identified process would likely benefit from a different type of instructional support. For example, novices were unlikely to independently discover a rule specific to elbow radiograph interpretation (e.g., mid radial line). Instead, learning these rules requires explicit didactic instruction and the opportunity to decide when to use *rule application* when working on varied cases (Iyer et al. 2012). By contrast, the process of *gestalt interpretation* will be most effectively developed by mastering a designed sequence of exemplars and foils aided by immediate and detailed feedback (Norman et al. 2007; Bruno 2018). The experts were able, in our study and others (Azevedo et al. 2007; Morita et al. 2008), to shift between methods in order to achieve superior performance. Consequently, the fact that the novice must learn each process, differentially invoked across cases, and additionally learn which applies when, speaks to the need to carefully engineer learning environments tailored to the specific needs of a domain of expertise.

Our research complements prior research by capturing more detail on the cognitive processes involved with trainees’ learning to perform a diagnostic skill accurately and may be used to design more effective training of specific cognitive processes associated with higher accuracy (Table 4).

The process of *gestalt interpretation* is difficult for an expert to teach a trainee, because experts often report that it is a matter of pattern-recognition, where there are no reportable intermediate steps (Ericsson and Simon 1993; Norman et al. 2007). It is clear that a complete novice is helped by an initial description but with more and more practice the completion of the process requires hardly any attention and is completed rapidly and effortlessly (Fitts and Posner 1967). The development of the effortless pattern recognition requires a large number of examples (and non-examples) and certainly an order of magnitude more cases than provided in our study (Boutis et al. 2016; Norman 2009; Taylor 2007)

The acquisition and refinement of *purposeful search* is very likely to benefit from direct instruction. For example, learning to characterize the fat pads, which are differentially predictive of fracture, using sematic qualifiers (small, medium and large) across a range of specific examples could help improve a learners’ overall diagnostic accuracy. Such part-task practice could apply to identifying any number of visual features, how they vary with age and, importantly, how to identify a pertinent negative. From our analysis of the protocols it appeared that the novices were learning a more purposeful search but in a haphazard, inefficient manner. One would expect that many more and different examples would be necessary to conceive of the range of appearance of fat pads, to name only one component of a purposeful search.

On the surface, *rule application* seems to be the theme that best lends itself to intentional didactic approaches; however, even here we note a role for targeted repetitive practice. The experts did not apply every rule to every case but instead had learned to invoke the rule preferentially in the cases where their perception was that it could help differentiate. It may be that the novices need to both learn the rule and then learn with case exposure when it does NOT apply, a process that appeared tacit in the verbal protocols.

Novices verbalized the use of *knowledge from a prior case* more often than experts. Based on the verbal protocols (Table 9 in “Appendix”) the novices appear to be using a learning-by-comparison method: the current case against one still present in recent memory (Kok et al. 2015; Beckstead et al. 2017). This is suggestive of benefits of a deliberate instructional design—for example, making it easy for learners to compare their accumulated cases side-by-side, as well as sequencing cases so that early cases are prototypical (and comparable), serving as foundational exemplars. Prior research suggests that experts likely compare the present case, not to prior cases in our program, but instead to well-encoded representations accessed from long-term memory (Norman 2009; Norman et al. 2007; Ericsson and Kintsch 1995). They may have been relatively unaware that they were mapping from long-term memory and, in turn, may not have been able to vocalize this during the study (Norman 2009).

These results suggest that, instead of leaving the trainee to the mercy of self-guided study or the idiosyncratic mix of cases that present to a clinical service, the educator should instead present an intentional choice and order of cases that explicitly serve to strengthen the trainee’s cognitive representation (Ericsson 2018b). The four themes identified in this study could also be considered through the lens of dual process theory which describes System 1 and System 2 thinking (Kahneman 2002). The *gestalt interpretation* we identified is an example of System 1 thinking and, that this behaviour was more frequent in experts, is entirely consistent with what has been previously reported in radiology and expertise literature (e.g. van der Gijp et al. 2017; Morita et al. 2008; Norman et al. 2017). On the other hand, the *purposeful search* aligns more with System 2 thinking. This was used more frequently in normal cases since in abnormal cases identification of the abnormality typically halted the search, while the end of the searching strategy for normal cases was often less certain. *Purposeful search* was also used more frequently by experts. It may be that novices lack the fundamental, fine-grained knowledge to apply the necessary elements of this strategy. This aligns with the research by Sherbino et al. (2014) which demonstrated that instructing medical students in use of system 2 strategies did not reduce their biases in diagnostic reasoning. In our study, the decision to apply a rule could also reflect System 1 thinking, even though rules themselves are largely analytical in nature. For example, in the cases where the diagnosis is obvious (e.g. a well demarcated Type III supracondylar fracture), applying the analytical rule would add little to the System 1 radiograph interpretation. Thus, a key characteristic of expertise in our study was the ability to invoke both System 1 and System 2 strategies, as needed. Future research could use a greater number of more specific case prompts to closely examine the emergence in trainees of more accurate balance between the mechanisms we have delineated.

Our study has limitations that warrant consideration. While each verbal protocol yielded a rich description of the active thought processes of the participant, we had relatively small numbers of participants doing a relatively small number of cases, which is typical for studies employing protocol analysis. The motivation to perform well on our tasks was likely different between the experts and novices in that the reputational risk for novices is quite small compared with that for experts, even in the research context. We only examined one specific radiograph type in this study so as to generate case-to-case variability that is within the bounds of one educational context. Our findings must therefore be generalized cautiously to other contexts based on the degree to which our findings are consistent with the more general literature across other types of visual diagnosis. The coding of our think aloud protocols was done by a single coder with expertise in radiology interpretation, cognitive science and coding of textual data. His analyses, while blinded to the expert-novice status of the participants, were corroborated by quantitative verification of expert-novice differences in the thematic codes assigned, but other methods of triangulation were not used. Our results were based on the sufficiency of accounts of participants’ verbalized thoughts’ mediation of their performance but does not make inferences about information that was not reported (Ericsson and Simon 1993; Norman 2018). Not all cognitive processes can be accessed by this method.

In summary, using the protocol analysis of think-aloud verbalizations, we have demonstrated how the cognitive processes of novices who are learning compare with experts during radiograph interpretation of a sequence of images. These processes included *gestalt interpretation*, *purposeful search*, *rule application*, and *reasoning from a prior case*. We were able to find evidence that these processes could be associated with accuracy of the diagnoses. All processes except *reasoning from a prior case* were applied more frequently by our experts. *Gestalt interpretation* was also used with higher frequency in abnormal cases, while *purposeful search* was used more often for normal cases. Our findings provide guidance for the design of deliberate practice that uses well-curated image banks and analytics to facilitate the novice’s journey to expertise in image interpretation.

## Appendix

See Fig. 4 and Tables 4, 5, 6, 7, 8 and 9

**Table 4:** Radiograph collection and calibration process

*Collection of elbow radiographs* Paediatric elbow radiographs have been selected for this research. Elbow injuries are common in children and are relevant for a wide variety of physician disciplines such as emergency physicians, paediatricians, and paediatric emergency physicians. Also, this type of image has been found to be difficult to interpret and may even lead to unnecessary imaging (e.g. obtaining radiographs of the uninjured elbow and the injured elbow to delineate what is ‘normal.’), and there are potentially high stakes if a physician makes an error in radiograph interpretation. In very young children, an elbow fracture identified on radiographs may be the only clue to child abuse which, if missed, exposes the child to life-threatening consequences. Finally, elbow fractures in children have one of the highest potentials for associated neurovascular compromise. Sequelae may include long-term contractures and limited mobility. We collected the elbow radiographs by purposively identifying them from the Paediatric Emergency Department (ED) at the Hospital for Sick Children, Toronto, Canada. In our prior research we have learned the importance of consecutive collection of media from the clinical environment, to ensure an appropriate spectrum of illness. We identified all elbow radiographs that were taken over a two year period for the purpose of excluding pathology, about 400 cases. At the Hospital for Sick Children, there is an electronic database that records all radiographs ordered and access to this is available through the medical records database team. A query was carried out by the medical records database team returned a list of all the relevant radiographs done in the emergency department in the two years preceding the start of this research. The list of these radiographs, associated patient histories and demographics, and their respective radiology reports was organized into an Excel spread sheet by a research assistant. These radiographic images and corresponding patient data were then be reviewed in detail by two paediatric emergency medicine physicians. From this set of films, we excluded the following cases: all radiographs that had embedded markers (e.g. arrows) that suggest a diagnosis, very poor quality films such that radiographic findings are obscured and radiographs where a consensus final diagnosis could not be obtained. From this resultant pool of radiographs, two paediatric emergency physicians and a consultant radiologist selected 328 elbow radiographs which provided the following: a frequency of abnormal/normal/normal variants radiographs consistent with that seen in actual clinical practice and cases that emphasize the necessary educational content for most paediatricians and emergency physicians. As such, single examples of rare normal variants/abnormal/controversial cases was (initially) retained while duplicate examples were removed. All cases were categorized as clinically normal or clinically abnormal based on the information provided by the official radiology report, and a review of the patient clinical information. A clinically normal radiograph was defined as a radiograph that does not have pathology that would normally lead to a change in patient management. A clinically abnormal radiograph was defined as a film with identifiable pathology (e.g. visible fracture, posterior fat pad sign) that commonly leads to a change in patient management. A change in patient management may include one or more of the following events as noted on the patient medical record: consultation with a subspecialist in the ED or in follow up, placement of immobilization, and/or admission to hospital. Clinically abnormal films were further sub-classified by diagnosis and the location of the abnormality on the image. *Radiograph calibration* Physicians of varying degrees of medical training interpreted the pre-screened 328 elbow radiographs. From two large research networks, we recruited 111 raters including fourth year medical students, residents, PEM fellows, and staff physicians in PEM and radiology. Participants completed a minimum of 80 cases which included a common set of 20. From this, we determined the index of discrimination and proportion correct (p) and retained items that performed well. The result was a set of 285 elbow radiograph with appropriate item difficulties and relevant education content. The study radiographs were chosen from this pool according to the process described in the main text.

**Table 5:** Preliminary Codes

Code	Description	Ultimate Theme
*Codes from published protocols for interpretation of pediatric elbow radiographs* *Jacoby* 2007, *Iyer* 2012		
Positioning	Is the arm positioned properly so that all features can be identified?	
Fat pads generally	Did the participant comment on the fat pads in general or specifically name one or both?	Rules
Anterior fat pad		
Posterior fat pad		
Alignment generally	Did the participant comment on the alignment of the bones or more specifically invoke the specific lines?	
Anterior Humeral Line		
Radiocapitellar Line		
Radial Head Dislocation	Identify specific diagnosis	
Ossification Centers	Specify these specific structures	
Fracture—GENERAL SCAN	Did the participant carry out a general scan of the bony structures without name specific anatomic features?	Purposeful search
Fracture—Supracondylar	Did the participant, by name, specify either looking for or finding these specific fracture types?	
Fracture—Lateral Condyle		
Fracture—Medial Epicondyle		
Fracture—Olecranon-Ulna		
Fracture—Proximal Radius		
Fracture—Transphyseal		
Normal variant	Did the participant diagnose a specific radiograph finding as being within the range of normal, not requiring intervention?	
*Emergent codes*		
History	Does the participant read the history from the screen?	
History—Concordance	Comment on concordance of history with the radiological findings observed?	
Soft-tissue swelling—presence		
Soft-tissue swelling—concordance	Concordance of soft-tissue swelling with the location or other feature of any observed radiological lucency?	
Confirm in two views	Did the participant confirm the presence of a radiological feature in two separate views?	Purposeful search
Orientation—medial v lateral	Comments on whether a feature is medial or lateral anatomically	
Maturity	Comments on the level of skeletal maturity of the child; has a sense of the normal progression	
Prior case	Comments on a prior case with respect to interpreting the current one	Prior case
Mineralization	Comments on the degree of mineralization of the bones	
Decision-making	Comments on their process for deciding whether a particular feature is significant	
Confidence	Comments on their confidence in their decision or diagnosis	
Zoom-Windowing	Describes the action of zooming in on a finding	
Prototype in memory	Alludes to a mental prototype and the deviation of the current film from that prototype	
Order of Identification	Which parts of the radiograph were attended to in what order?	Gestalt

**Table 6:** Gestalt theme. Undisplaced fracture of the proximal ulnar bone (at red circle). The yellow square represents the mask designating the correct location of the fracture. (Color table online)

Correct	Correct	Incorrect
“AP view radiograph of the right elbow. It’s five-year-old female with right elbow injury, marked swelling and pain of right elbow. The proximal ulna looks a little bit abnormal. I‘m seeing streaky lucencies through it.” [Exp-Vandan]	“The next case is a five-year-old female with right elbow injury, uh, mark swelling and pain of right elbow. So, I’m looking at the AP view here and, uh, what I’m seeing is, uh, some linear lucencies through the proximal ulna.” [Exp-Vluck]	“Okay, so five-year-old female with right elbow injury; marked swelling and pain of right elbow. There’s something floating [wrong feature, radial ossification center], here. That doesn’t look good. And I can’t see it on this view, so I’ll put the floating thing as not right. [Novice-Vgere]
Displaced fracture of proximal ulna	Displaced fracture of proximal ulna	Displaced fracture of proximal ulna
Expert	Expert	Novice

**Table 7:** Purposeful search. Normal elbow x-ray in an 11 year old boy. Ossification centers and growth plates result in features that have to be considered as possible fractures

Correct	Correct	Correct
“This is an 11-year-old male. Uh, fell while on slide. I’m looking at an AP view of the right elbow and I am looking at the bones to see if I can find any, uh, irregularities, cortical irregularities or any fracture lucencies. Um, I do not see anything, um, that’s striking at the moment. I, of course, wanna see the lateral view. I do not see elevation of the anterior or the posterior fat pads so there is no elbow joint effusion here. I don’t see any areas of lucency that would suggest a fracture. I don’t see any areas of cortical irregularity to suggest a fracture so I would say this is definitely normal” [Exp = Vluck] D0039	11-year-old male fell while on slide. I think this might be an area of fat, but it might just be a fat person because it’s not bulging in one spot. And it looks like there’s something in the joint space, but this alignment looks normal. This is where it has to be an olecranon. Let’s check—I can’t really tell. I’ll have to say this is normal. [Novice-Vgere] D0039	Okay, there’s a fracture extending through the proximal shaft to the ulna and to the articular shaft is a little rougher on the fact [inaudible] [00:15:35]. 11-year-old male fell on slide, okay, so again, almost based skeletally mature but I still see some open physes. This really overlaps so I’m not seeing this area very well so I definitely wanna check it out on the lateral view. Okay, I’m turning my head because I’d like to see a line little bit the other way but I’m not seeing necessarily an anterior fat pad. I’m not seeing a posterior raised fat pad per se I see the olecranon here is not completely fused and let’s see, anything else here? Other bones seem intact. This is not so helpful. This looks well-corticated so I don’t think that’s a fracture. I think that’s another ossification center then just catching a certain part of these lines look like they are fairly well-corticated. I’m really not seeing definite abnormalities so I wanna say this probably normal, see what happens
Normal Case D0039	Normal Case D0039	Normal Case D0039
Expert	Novice	Expert

Example of Coding: “There may be a small [SM] joint effusion because the anterior fat pad is elevated. Again, I don’t see significant [SM] soft tissue swelling; it looks like an appropriate[SM] lateral view. I don’t see [PN] the posterior [SM] elevation of the fat pad. I think that I would just go back to the AP view to check [PN] the capitellum [V] as well as the radial head [V].”

*SM* semantic modifier; *V* vocabulary; *PN* pertinent negative

**Table 8:** Rule application. Elbow dislocation in an 11 year old boy. There are no fractures resulting in disruption of the bony cortex or jagged edges. Instead, the radial head is dislocated which can be diagnosed with certainty by drawing the Mid-Radial Line (MRL) which should normally intersect the Capitellum of the humerus

Incorrect	Correct	Correct
“Caught and twisted arm in a slide. There’s something overlaying the elbow, I’m not sure if that’s a seat or not, but looking at the lateral view. Okay, there’s something overlaying it. I’m not sure what it is. It does—looks like—this looks—strange opacities in the distal humerus, I’m not sure what it is, but I don’t see any radiolucencies around the elbow so I don’t think there are any joint effusions. There’s a little bit of very mild soft tissue swelling in the anterior aspect of the elbow, but otherwise, I don’t—the periosteum looks intact. I don’t see any step-offs. So, I’m gonna go with probably normal.” [Exp-Vdist]	“This was a seven-year-old male who caught and twisted his arm in a slide. We’re looking at the AP view, and just already right off the bat I feel like it looks like it’s out of alignment. It’s a little disorienting because it’s kind of horizontal compared to how it usually is So I’m gonna go ahead and look at the lateral view. I just feel like the elbow is malaligned from how it should be. And especially given this kind of, like, twisting injury, I think that would be kind of consistent with some type of dislocation. And to me the thing that looks most abnormal is the ulna, so I’m gonna say that’s abnormal.” [Novice-VBRED]	“Okay, next case is a 7-year-old male caught and twisted arm in a slide. So we have, um, a—a view of the elbow, um, positioned somewhat unusual, uh, to what I’m accustomed to. Um, it looks like it’s an AP view but the patient—but the—the x-ray is rotated 90 degrees and so, um, uh, it looks to me like the—the radial capitellar joint is off. When I draw my line, there’s anterior, um, dislocation of the radius relative to the capitulum. I don’t see any obvious fracture I’m now gonna look at the lateral view. Um, again, I can see that the joint is deranged here. There’s a small joint effusion and, um, this looks like a dislocation, um, anterior dislocation of the, um, the radius. So this is definitely abnormal.” [Expert-VLUCK]
Radius dislocation Expert fails to invoke MRL and incorrect	Radius dislocation Novice fails to apply MRL rule; is correct but uncertain	Radius dislocation Applies MRL rule; is certain.
Expert	Novice	Expert

**Table 9:** Prior Case

VPREN-D0190 The learner diagnoses the new case (LEFT) based on remembering a previous case (RIGHT) wherein the posterior fat pad had been declared normal/negative. (carries in memory the previous case in order to make sense of the new case). Experts did not vocalize such comparisons		
I think this one [PICTURED-LEFT] is probably abnormal because last time they said it was okay because there was no posterior fat pad [PICTURED-RIGHT]. But now there is a posterior fat pad, or maybe that’s an effusion.	“The first thought I remember thinking was that last case. I want to look a little bit closer on this one to try to identify any specific fractures I would otherwise miss.”	Similar to that case, two or three cases ago, there’s a little bit of abnormality here along the lateral condyle.
VPREN; D0190	Vandan 287; D0350	Vandan D0364
Initial look at the …. is very different than the last one, much more mature looking joint structures	Again, in this one I would say there is an absence of a fat pad sign, but I’m getting more and more worried about saying that given 2 of the previous cases	There’s this little floating thing I saw in the first case, and they didn’t mention anything about that, so I think that’s fine as well
VNUGH 037; D0001	VNUGH037; D0100	VPREN; D0374
“I remember trying to identify a fracture and I know my first thoughts were trying to identify a fracture because, well, it was what we had on the last case. I didn’t see that on this case”	I’m going to look for any splits in the surface of the bone, which like in the previous one, I was surprised a little at how small it was so I’m going to try to be more careful on this one	So, the first thought I had was that this kind of looked like the kid from before, except for the tilt in the humerus because the big space didn’t mean anything for the first kid
Vandan 287; D0001	VCLAR; D0100	VGERE; D0100
